# An Account of the Salutary Effect of Blisters in a Case Attended with Symptoms of Hydrocephalus Internus

**Published:** 1782

**Authors:** 


II. *An account of the salutary effect of Blisters in a case attended with symptoms of Hydrocephalus internus. By* Robert White, M. D. *Physician at Bury St. Edmunds. Read* Nov. 4, 1782.

A Few weeks ago I was called to the son of Thomas Garwood, coach-maker in Bury St. Edmund's. This boy is about four years old, of a pale complexion, and weak habit, in consequence of being badly nursed. He had complained for several days of great pain and weight in his head, which, when I first visited him, he could scarcely bear to have raised from the pillow: but what gave the greatest alarm to his parents was, a total blindness, which had gradually supervened. His pulse was irregular, and sometimes remarkably slow. He was several times seized with universal thrillings and twitchings, such as generally precede convulsions; after which came on a slight degree of fever, attended with immoderate thirst. His body was rather bigger than its natural size, very tense, and had been for some time inclined to costiveness. Upon examining his eyes, I found them drawn obliquely towards the nose, and the pupils dilated to nearly the size of the cornea, without the least power to contract.

Con-

Convinced that this dreadful affliction proceeded either from worms, or from water in the head, my aim to relieve was twofold. I therefore ordered a large blifter to be applied directly to his head, and the nape of the neck; and the following purge to be taken early in the morning:

℞ Pulv. rhæi opt. gr. xij.

Mercur. dulc. gr. ij.

Ol. rutæ, gutt. j. M.

I faw him that morning about ten o'clock, and found him much relieved from pain, and fitting upon his mother's lap. The blifter had difcharged moft profufely throughout the night. The purge had no other effect than making him retch once or twice about the middle of the day. In the evening he was more revived, and the pupils began vifibly to contract. I ordered the purge to be repeated the next morning. That day, being the third of my attendance, he gave feveral proofs of returning fight, and was in tolerable fpirits. The fecond purge gave him four loofe ftools, which evacuation left him extremely languid. On the fourth day he took nothing but light folid food, which he ate rather greedily. His eyefight was much recovered, being able to diftinguifh fome moderately-fized objects. He alfo ventured to move about the room, and his ſpirits were more regular. His belly was ſtill præternaturally enlarged, but by no means ſo tight as before. I now preſcribed the following powders:

℞ Stanni pulv. ℈j.

Sem. ſanton. gr. vj.

Ol. rutæ, gutt. ſs. M.

One of theſe was taken twice or thrice a day, and the purging powder repeated once in five or ſix days.

On the ſixth day the bliſter was renewed at the hind part of the head. The patient continued to mend daily; and in the ſpace of a fortnight from that time was in much better health than before this complaint ſeized him. I then ordered an infuſion of bark with the *tinct. flor. martial.* to be taken twice a day. His eyes have not quite recovered their natural poſition; but the pupils regularly contract, and he ſees, to all appearance, as well as ever.

I had ſome thoughts at firſt of rubbing in mercurial ointment, but obſerving the favourable change on the morning of the ſecond day, I was unwilling to preſcribe it.

I think we may venture to declare, that this boy's diſorder was occaſioned by a depoſit of lymph in the cavities of the brain; and that the cure cure was principally, if not totally, produced by the application of the blifter, which for the firft four days difcharged moft plentifully.

I never could obferve any particular increafe of the fecretions, except a flight flux of pale urine, in the night after the operation of the fecond purge.

No worms, or fymptoms of them, ever appeared, although the purge, with the addition of *mercur. dulc.* gr. j. was repeated twice more at proper intervals; and the vermifuge powder was taken two or three times a day for a fortnight.

*Bury St. Edmunds,*

*Oct.* 7, 1782.

